# Glomerular Organization in the Antennal Lobe of the Oriental Fruit Fly *Bactrocera dorsalis*

**DOI:** 10.3389/fnana.2018.00071

**Published:** 2018-08-29

**Authors:** Tao Lin, Chaofeng Li, Jiali Liu, Brian H. Smith, Hong Lei, Xinnian Zeng

**Affiliations:** ^1^Guangdong Engineering Research Center for Insect Behavior Regulation, South China Agricultural University, Guangzhou, China; ^2^School of Life Sciences, Arizona State University, Tempe, AZ, United States

**Keywords:** antennal lobe, glomeruli, olfaction, digital atlas, *Bactrocera dorsalis*

## Abstract

The oriental fruit fly, *Bactrocera dorsalis* is one of the most destructive pests of horticultural crops in tropical and subtropical Asia. The insect relies heavily on its olfactory system to select suitable hosts for development and reproduction. To understand the neural basis of its odor-driven behaviors, it is fundamental to characterize the anatomy of its olfactory system. In this study, we investigated the anatomical organization of the antennal lobe (AL), the primary olfactory center, in *B. dorsalis*, and constructed a 3D glomerular atlas of the AL based on synaptic antibody staining combined with computerized 3D reconstruction. To facilitate identification of individual glomeruli, we also applied mass staining of olfactory sensory neurons (OSNs) and projection neurons (PNs). In total, 64 or 65 glomeruli are identifiable in both sexes based on their shape, size, and relative spatial relationship. The overall glomerular volume of two sexes is not statistically different. However, eight glomeruli are sexually dimorphic: four (named AM2, C1, L2, and L3) are larger in males, and four are larger in females (A3, AD1, DM3, and M1). The results from anterograde staining, obtained by applying dye in the antennal lobe, show that three typical medial, media lateral, and lateral antennal-lobe tracts form parallel connections between the antennal lobe and protocerebrum. In addition to these three tracts, we also found a transverse antennal-lobe tract. Based on the retrograde staining of the calyx in the mushroom body, we also characterize the arrangement of roots and cell body clusters linked to the medial antennal-lobe tracts. These data provide a foundation for future studies on the olfactory processing of host odors in *B. dorsalis*.

## Introduction

The oriental fruit fly *Bactrocera dorsalis* is an important pest of vegetables and fruit (Drew and Hancock, 1994), causing huge damage to agricultural products through larvae feeding. This insect feeds on over 250 species of plants, and is listed as a dangerous invasive species in many countries (Clarke et al., 2005; Stephens et al., 2007). Population monitoring is therefore a focal point of controlling strategies against this insect. Methyl eugenol (ME) is an effective attractant to this insect, which has been widely used together with insecticides for monitoring and managing purposes (Fletcher, 1987; Shelly et al., 2004; Tan and Nishida, 2012). However, ME only attracts adult male flies (Tan and Nishida, 2012; Liu et al., 2017), limiting its power in monitoring the entire fly population. Additionally, ME is considered as a carcinogen to humans (Smith et al., 2002), calling for a need to develop safer and more efficient attractants. One example comes from γ-octalactone, a volatile from mango fruit, which has been reported to elicit strong oviposition responses in *B. dorsalis* (Damodaram et al., 2014; Jayanthi et al., 2014). Olfaction plays a crucial role in the behavior of *B. dorsalis*. To develop more or better attractants and repellents, detailed knowledge about the anatomy of the olfactory system of this insect is necessary.

Reverse neuroethology, a strategy for identifying behaviorally significant stimuli from studying the neurobiology of a species, could offer a new way to develop attractants or repellents. Many behaviorally critical volatiles, such as sex pheromones in moths, are processed in a specialized region of the olfactory center of the brain (Hansson et al., 1991; Lei et al., 2013; Berg et al., 2014). Findings on such evolutionarily formed environment-brain links inspire research on the anatomy of insect central nervous systems, especially in combination of with neurophysiological techniques such as calcium imaging and multiunit recordings, which allow observation of odor-evoked responses simultaneously across multiple brain regions (Lei et al., 2004; Linz et al., 2013; Trona et al., 2013; Byers et al., 2014; Wu H. et al., 2015; Bisch-Knaden et al., 2018). Toward this goal, we investigated the anatomical structure of the primary olfactory center, antennal lobe (AL), of the oriental fruit fly in both sexes.

Natural odorants are recognized by olfactory receptors (ORs) that are located on the dendrites of olfactory sensory neurons (OSNs) on antenna or other appendages (Vosshall et al., 2000; Couto et al., 2005). OSNs project their axons into an array of neuropil modules in the antennal lobe (AL), called glomeruli, where the OSNs are synaptically connected with the projection neurons (PNs) and local neurons (LNs). The AL is functionally analogous to the olfactory bulb in vertebrates, and represents the primary olfactory central neuropil for odor processing (Homberg et al., 1989; Hildebrand and Shepherd, 1997; Hansson and Anton, 2000; Schachtner et al., 2005). In many insect species, it has been shown that the axons of OSNs expressing the same OR converge onto mostly one glomerulus (Gao et al., 2000; Vosshall et al., 2000; Couto et al., 2005), suggesting that the size of a glomerulus may be correlated with the number of cognate OSNs, which in turn may reflect the importance of the encoded odor stimuli.

Sexual dimorphism in the AL can also provide valuable insights into the olfaction-mediated behaviors of insects. In Lepidoptera, the male AL often has a set of enlarged glomeruli, namely a macroglomerular complex, for processing information on female sex pheromones (King et al., 2000; Hansson et al., 2003; Wu H. et al., 2015). The female AL, on the other hand, possesses specialized glomeruli for odor cues related to oviposition (King et al., 2000; Rospars and Hildebrand, 2000; Bisch-Knaden et al., 2018). A sexually dimorphic AL has also been reported in cockroaches (Watanabe et al., 2010), fruit flies (Kondoh et al., 2003), honeybees (Nishino et al., 2009). It is not known yet if *B. dorsalis* also shows sexual dimorphism.

Glomerular structure is a hallmark of chemotopical coding scheme. In the cross-fiber coding hypothesis, one odor activates multiple glomeruli, and one glomerulus can be activated by different odors (Knaden et al., 2012; Clifford and Riffell, 2013; Galizia, 2014; Haverkamp et al., 2018), which is hypothesized to have advantages of increased coding capacity and plasticity. In contrast, the labeled-line coding hypothesis states one-receptor—one—glomerulus principle, facilitating coding accuracy and speed (Lei et al., 2005; Reisenman et al., 2011; Chaffiol et al., 2012; Haverkamp et al., 2018). These two coding mechanisms likely co-exist in most insect species, based on reports on glomerular organization in honey bee (Galizia et al., 1999), parasitoids wasps (Smid et al., 2003), fruit fly (Laissue et al., 1999; Grabe et al., 2015), mosquitoes (Ignell et al., 2005; Ghaninia et al., 2007), and moth (Kazawa et al., 2009; Zhao et al., 2016). Characterization of glomerular organization is fundamental for future functional studies. To our knowledge, this is the first report on the anatomical characterization of AL in *B. dorsalis*.

## Materials and methods

### Insect colony

The *B. dorsalis* colony was established in 2010 in the Department of Pesticide Science, South China Agricultural University, Guangzhou, China. Adult flies were reared in cages (30 × 30 × 30 cm) and fed with an artificial diet (3:1 sugar to hydrolyzed yeast) and water. Larvae and pupae were kept at 25–27°C, 60–80% RH, and 14:10 h (L:D) photoperiod until adult flies emerged. Adults 4–6 days old were used in this study.

### Mass staining of antenna, maxillary palp, AL, and calyx

First, we carried out anterograde fills from the antenna and maxillary palp nerves following Zhao et al. (2016). The flies were prepared as described in Liu et al. (2015). Briefly, adult flies were secured in a 1-mL plastic tube so that only the head capsule was exposed. The head capsule was then fixed using wax (Kerr Utility Wax Rods, #09731). One antenna or the maxillary palp was cut off from the base and crystals of the fluorescent dye Micro-Ruby (TMR; tetramethylrhodamine dextran with biotin, Micro-Ruby, Molecular Probes; Invitrogen, Eugene, OR) were then placed on the cut end using a needle. Second, to visualize the antennal lobe tracts, we followed Tanaka et al. (2012b) with minor modification. The flies were fixed as described above, a window between the two compound eyes was cut with a razor blade. Muscles and tracheae were removed to expose the AL. Subsequently, a glass electrode coated with crystals of TMR was inserted into the AL and kept in this position until the dye was dissolved. Then, the electrode was dislodged; the brain was washed with saline to remove the extra dye. Third, for retrograde staining of the PNs, flies were decapitated, and the posterior head capsule was removed to expose the calyces, then a tapered glass electrode coated with crystals of TMR was manually inserted into the mushroom body calyx. After the dye was dissolved, brain was washed with saline to remove the extra dye.

After dye injection, the insects were placed in a dark and moisturized chamber at 4°C for 6 h or overnight. Insects were then decapitated, and brains were dissected out in Ringer's saline (in mM: 150 NaCl, 3 CaCl_2_, 3 KCl, 25 C_12_H_22_O_11_, and 10 TES buffer, pH 6.9) for synapsin immunostaining or confocal observation as described below.

### Immunohistochemistry

In order to visualize individual glomeruli within the AL, anti-synapsin antibody staining of the neuropil structures was performed. We followed Zhao et al. (2016) with minor modifications. Briefly, flies were decapitated, and brains were dissected in Ringer's saline solution. The dissected brain was fixed in 4% paraformaldehyde in 0.1 M phosphate-buffered saline (PBS, pH 7.4) overnight at 4°C. Subsequently, the brains were washed 4 times 20 min each in PBS with 1% Triton X-100 (PBST). After rinsing, preparations were pre-incubated in normal goat serum (NGS; Sigma, St. Louis, MO, USA; 5%) in PBST at room temperature for 3 h. The brains were then incubated in primary antibody SYNORF1 (Developmental Studies Hybridoma Bank, University of Iowa, dilution 1:100 in PBST containing 5% NGS), for 5 days at 4°C. Brains were then rinsed in PBST for 6 times (20 min, each) at room temperature, incubated in the secondary antibody, Cy5-conjugatedanti-mouse (Invitrogen, Eugene, OR; dilution 1:200 with 5% NGS in PBST), for 3 days at 4°C, washed 6 × 20 min in PBS, dehydrated with an ascending ethanol series (30, 50, 75, 95, 100% × 2, 10 min each), cleared in methyl salicylate, and mounted in Permount in a perforated aluminum slide with two glass covers lips.

### Laser scanning confocal microscopy

Stained samples were visualized with a laser scanning confocal microscope (LSM 780 META Zeiss, Jena, Germany) with Zeiss Plan-Neofluar 10 × /0.3 and 20 × /0.5l dry lens objective. A 633-nm line of the HeNe laser was used to excite the dye (Cy5) and a laser 543-nmline to excite the Micro-Ruby. The images were obtained with a resolution of 1,024 × 1,024 pixels in the *xy*-plane and an interslice distance of 0.6–1 μm.

### Three—dimensional reconstruction of glomeruli and ALTs

To make a 3D atlas of the glomeruli, we used Amira 5.4.1 (Visage Imaging, Fürth, Germany) to reconstruct confocal image stacks. The glomeruli and ALTs were labeled by using the segmentation module, including the “brush” and “masking” tools. Individual glomeruli volumes were measured with the MaterialStatistics tool, and imported to Excel (Microsoft) for further processing. Antennal and maxillary palp sensory neurons as well as antennal lobe tracts (ALTs) (and somata) were also rendered using the “projectionview” module in Amira.

Images were taken from single optical sections or from a complete stack by the camera of Amira software. Snapshots were further processed in Adobe Photoshop CS5 and Illustrator CS6 software (Adobe Systems San Jose, CA) for adjusting brightness and contrast, and text editing.

### Nomenclature of the AL glomeruli

Identification of glomeruli in the *B. dorsalis* ALs was based on their location, size and shape. The nomenclature used in this followed that for *Ceratitis capitate* (Solari et al., 2016) and *Drosophila melanogaster* (Stocker et al., 1990). Based on general position, the names of glomeruli start with one or two capital letters (A, anterior; P, posterior; D, dorsal; V, ventral; L, lateral; M, medial; C, central). To distinguish multiple glomeruli in the same region, we give every glomerulus a serial number followed the letters. Glomeruli identified across ALs were given the same numbers and colors in the 3D atlas.

We made a match of the position of each glomerulus in intra-individual (left and right) and inter-individual (female and male) ALs. Matching was achieved by comparing the confocal stacks directly at different levels of the z-axis throughout the AL and realistic 3D atlases. After matching the glomeruli in ALs, the volumes of the matched glomeruli were calculated. The neuropil structures of the brain were named as suggested by Ito et al. (2014), and the orientation of the brain refers to the axis of the insect body.

### Statistics

Base on the 3D atlas of AL, we can obtain the following parameters: volume of each glomeruli, overall volume of the AL (the volume of all stained glomeruli in a given AL). In order to examine the size differences of homologous glomeruli between the left and right AL, between individuals and between sexes, non-parametric *Mann-Whitney U*-test was performed. We also calculated the coefficient of correlation (r) of glomeruli size between individuals and sexes. All statistical analyses were performed using SPSS (version 20.0; IBM, USA). *P* < 0.05 were considered significant.

## Results

### General anatomy of the AL in *B. dorsalis*

**Figure 1:**
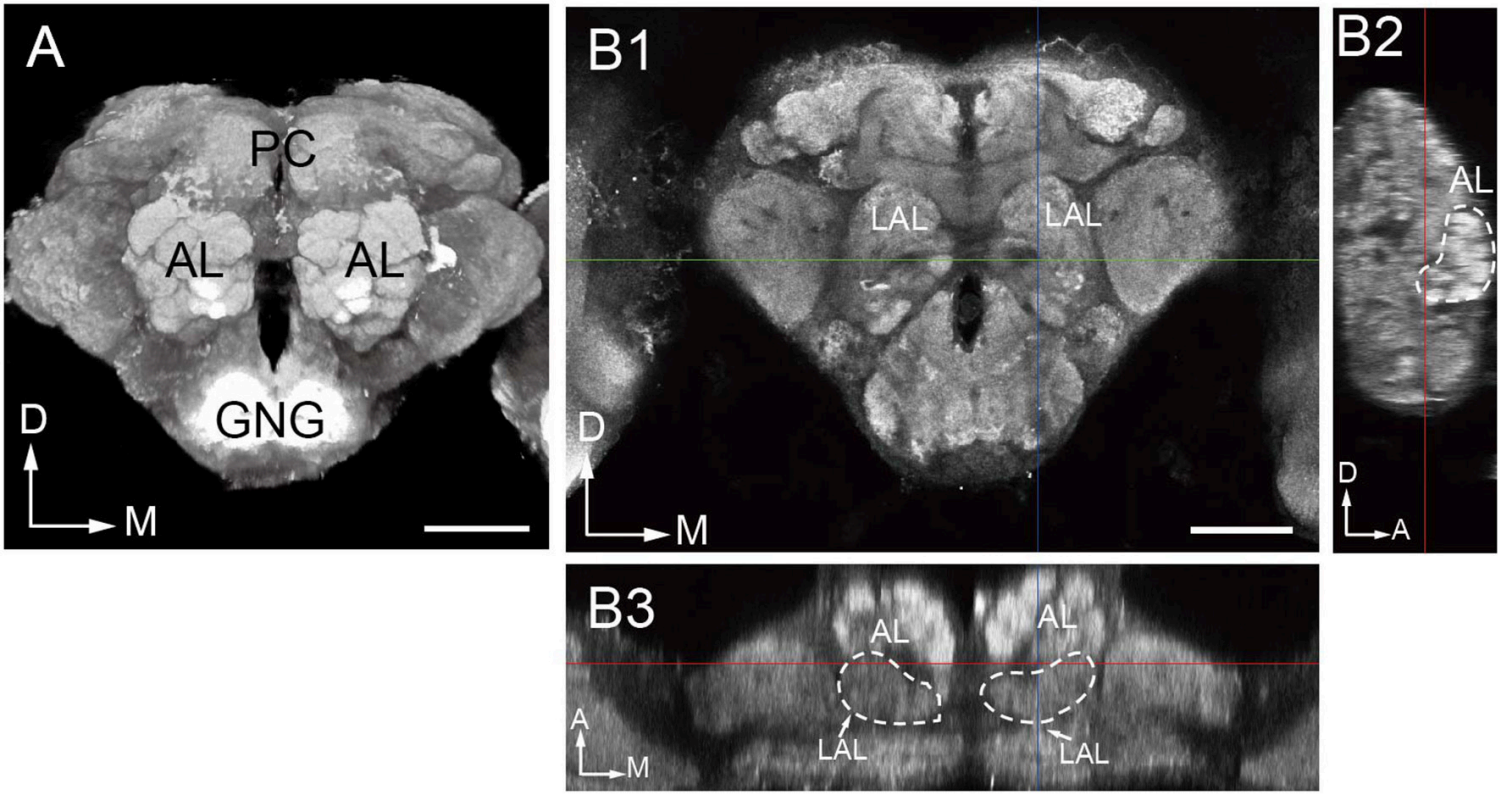
Overall structure of the oriental fruit fly brain. **(A)** Three-dimensional visualization of the image stack of a male brain, using direct volume rendering. The brain is shown in an anterior view, with the two prominent antennal lobes (AL) confining the numerous glomeruli. **(B)** Indication of the boundaries of the antennal lobe demonstrated by confocal images in frontal **(B1)**, sagittal **(B2)**, and dorsal **(B3)** views. LAL, Lateral accessory lobe; PC, protocerebrum; GNG, gnathal ganglia; D, dorsal; M, medial; A, anterior. Scale bar = 100 μm.

**Figure 2:**
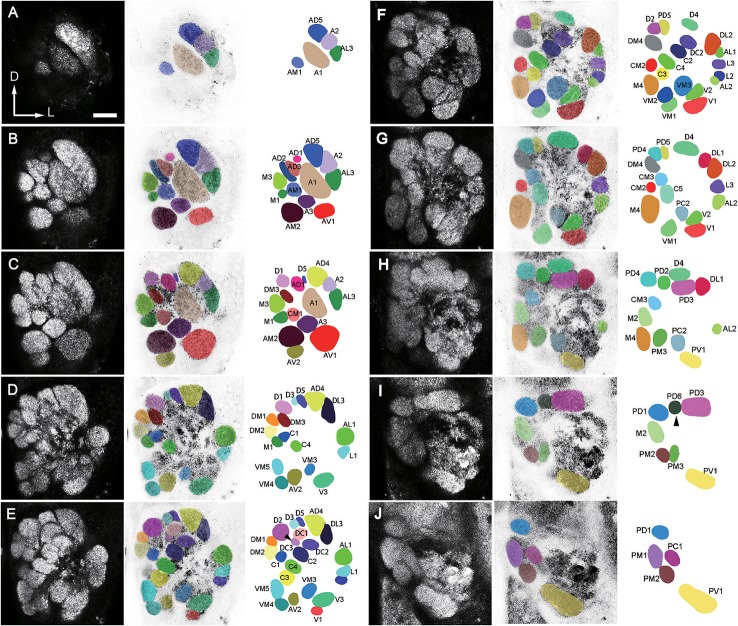
A confocal stack of the AL of a male *B. dorsalis* seen from anterior view. Ten sections from anterior to posterior at the depth of 4 μm in **(A)**, 9.6 μm in **(B)**, 15.2 μm in **(C)**, 21.6 μm in **(D)**, 27.2 μm in **(E)**, 33.6 μm in **(F)**, 39.2 μm in **(G)**, 45.6 μm in **(H)**, 51.2 μm in **(I)**, and 57.6 μm in **(J)**, were selected from a total of 89 images. The arrow head points to the missing glomeruli in some preparations. The orientation of the AL is indicated as: D, d**o**rsal; V, ventral; L, lateral; M, medial. Left column displays unaltered confocal images (raw data). Middle column displays demarcated glomeruli superimposed on negative confocal images. Right column displays identified glomeruli (for terminology see text). Scale bar = 25 μm.

Confocal sectioning of *B. dorsalis* brains allowed us to visualize the AL glomeruli in single image stacks. The ALs are located most anteriorly in the head capsule in relation to other brain structures, and are on the either side of the esophageal foramen (Figure 1A). Typically, the glomeruli are distributed in the outer layer of the AL surrounding a central fiber core devoid of any synaptic structure (Figures 1A, 2F–H). The posterior border of either AL is fused with the anterior end of the lateral accessary lobe (LAL) in the protecerebrum (Figures 1B, **9I**).

The dimensions of the whole glomerular area in x, y, and z axes are 147.23 ± 6.79, 157.89 ± 11.06, and 62.47 ± 3.09 μm in females (mean ± SD, *n* = 6), and 128.8 ± 7.39, 151.58 ± 12.24, and 64.58 ± 2.04 μm in males (mean ± SD, *n* = 6) (Table 1). Sixty-four or 65 glomeruli could be counted in each AL in females and males (*n* = 6), with a total volume of 301,550 ± 13,780 and 294,229 ± 10,062 μm^3^, respectively (mean ± SD, *n* = 6; Table 1).

**Table 1:** Volume and number of glomeruli.

**Preparation**		**Diameter (**μ**m)**			**Volume of all glomeruli (10^3^ μm^3^)**	**Number of glomeruli**	**Largest glomerulus (10^3^ μm^3^)**	**Smallest glomerulus (10^3^ μm^3^)**	**Mean volume of glomeruli ±SD (10^3^ μm^3^)**
		**X axis**	**Y axis**	**Z axis**					
Female	1R^a^	145.60	153.38	60.00	316.27	64	18.96	1.38	5.04 ± 3.09
	1L^a^	145.22	159.50	62.00	283.08	64	18.92	1.03	4.42 ± 3.00
	2R	135.54	146.33	68.45	314.33	65	16.00	1.19	4.88 ± 3.11
	2L	150.05	149.04	61.74	308.55	65	19.25	1.13	4.70 ± 3.08
	3R	152.76	176.80	62.44	298.58	64	16.95	1.06	4.66 ± 3.11
	3L	154.22	162.28	60.20	288.49	64	19.60	0.89	4.51 ± 3.02
Mean ± SD		147.23 ± 6.79	157.89 ± 11.06	62.47 ± 3.09	301.55 ± 13.78	64.3 ± 0.52	–	–	–
Male	1R	117.90	142.80	64.40	277.70	65	14.56	0.81	4.27 ± 2.91
	1L	126.75	151.25	65.27	296.43	65	17.37	1.00	4.56 ± 2.95
	2R	126.87	141.34	67.15	298.00	64	17.50	0.89	4.67 ± 2.84
	2L	127.04	141.09	66.07	308.60	64	17.00	1.38	4.90 ± 3.14
	3R	135.60	164.00	61.50	292.15	64	15.28	0.93	4.56 ± 2.85
	3L	138.67	169.00	63.12	292.48	64	18.30	1.20	4.57 ± 2.98
Mean ± SD		128.80 ± 7.39	151.58 ± 12.24	64.58 ± 2.04	294.22 ± 10.10	64.3 ± 0.52	–	–	–

a *R and L indicate the right and left antennal lobe, respectively*.

The number of identified glomeruli was consistent in two sexes (64–65, *n* = 12) and also within individuals. Most glomeruli could be identified across different individuals based on their position, shape and size, except for two glomeruli that were occasionally questionable. The DC3 glomerulus was not identifiable in both lobes of the F1 and F3 flies, and was the smallest glomerulus in the F2 fly (Figure 2E). The PD6 glomerulus was not identifiable in the left lobe of the M2 fly, and the right lobe of the M3 fly, and it was the smallest glomerulus in the M1 fly (Figure 2I).

### Identification of individual glomeruli

**Figure 3:**
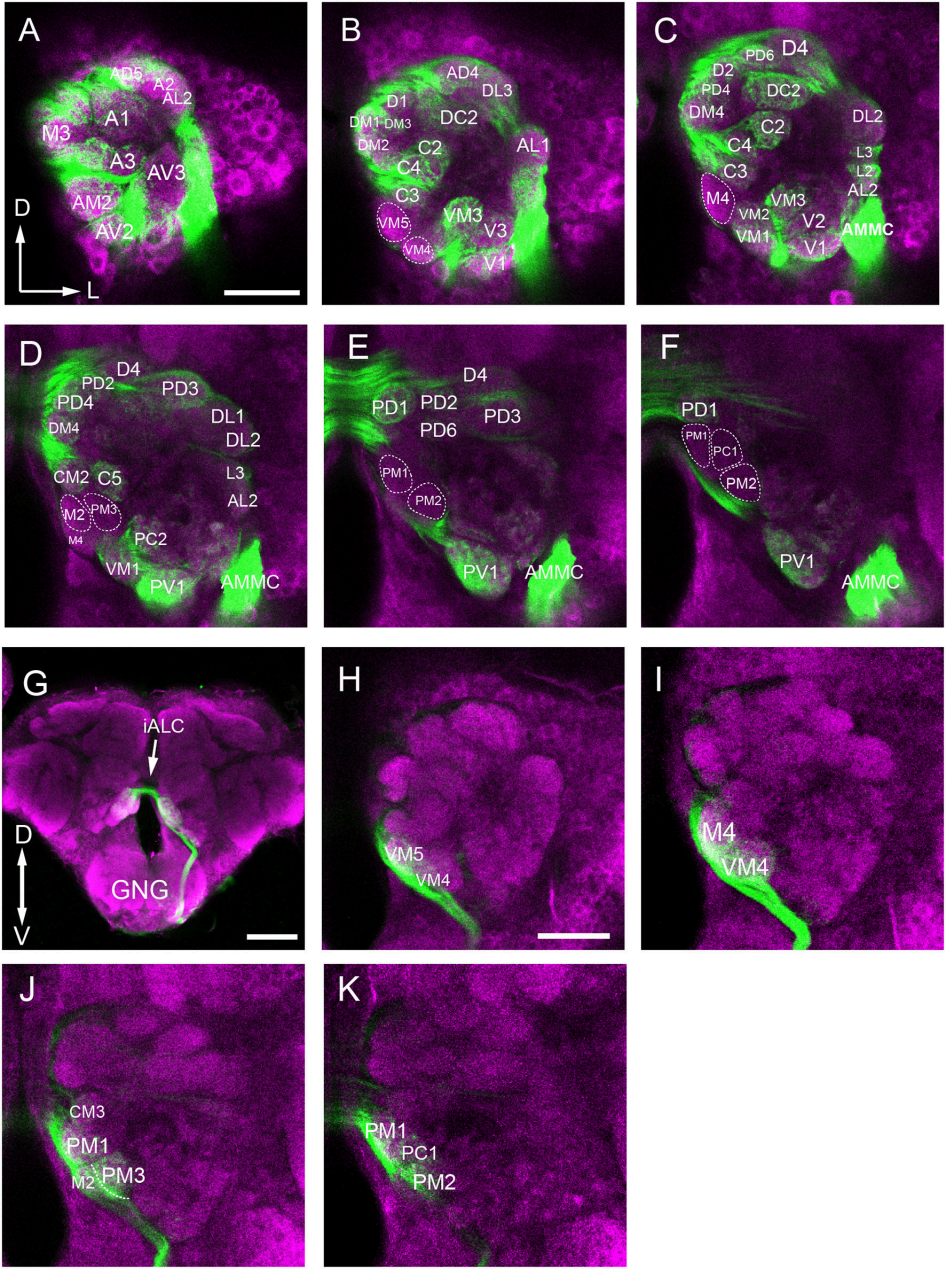
Confocal images showing OSNs from antenna and maxillary palp projecting into the AL. Labeling was obtained via anterograde mass staining of the antenna and maxillary palp sensory neurons. **(A–F)** Confocal sections at different depths of the AL showing glomeruli innervated by antennal ascending fibers. The overall brain outline (magenta) is marked by synapsin immunostaining. The most anterior glomeruli are shown at depth of 16 μm **(A)**, 23 μm **(B)**, 30 μm **(C)**, 38 μm **(D)**, 45 μm **(E)**, and 53 μm **(F)**. **(G)** The maxillary-palp nerve, via the gnathal ganglion, enters the medial part of the AL, and projects to the contralateral AL through the inferior AL commissure (iALC). **(H–K)** Sections of the AL showing glomeruli innervated by the maxillary palp sensory neurons. D, dorsal; L, lateral; M, medial; V, ventral. Scale bars = 50 μm in **(A,H)** (applies to **B–F**, **I–K**); 100 μm in **(G)**.

From 6 brains (3 for each sex) labeled with synapsin immunostaining, 6 were double-labeled with anterograde mass staining of OSNs (Figure 3), one AL was labeled via retrograde mass staining of PNs from the calyx in male (**Figure 9**).

The procedure used for identifying the individual glomeruli was similar to that used by Solari et al. (2016). For clarity, we divided the AL into six regions, i.e., anterior (A), dorsal (D), ventral (V), Central (C), lateral (L), and medial (M). Within each region, we first selected a few landmark glomeruli based on their shape, size, position, and demarcation. Then we identified the remaining glomeruli by their position relative to the landmarks. The identification of individual glomeruli from synapsin immunolabeling (Supplementary Video 1) was also confirmed by additional mass staining of OSNs from the antenna and the maxillary palp (Figure 3), and PNs confined to the medial antennal lobe tract (mALT) (**Figures 9D–I**).

#### Anterior region

**Figure 4:**
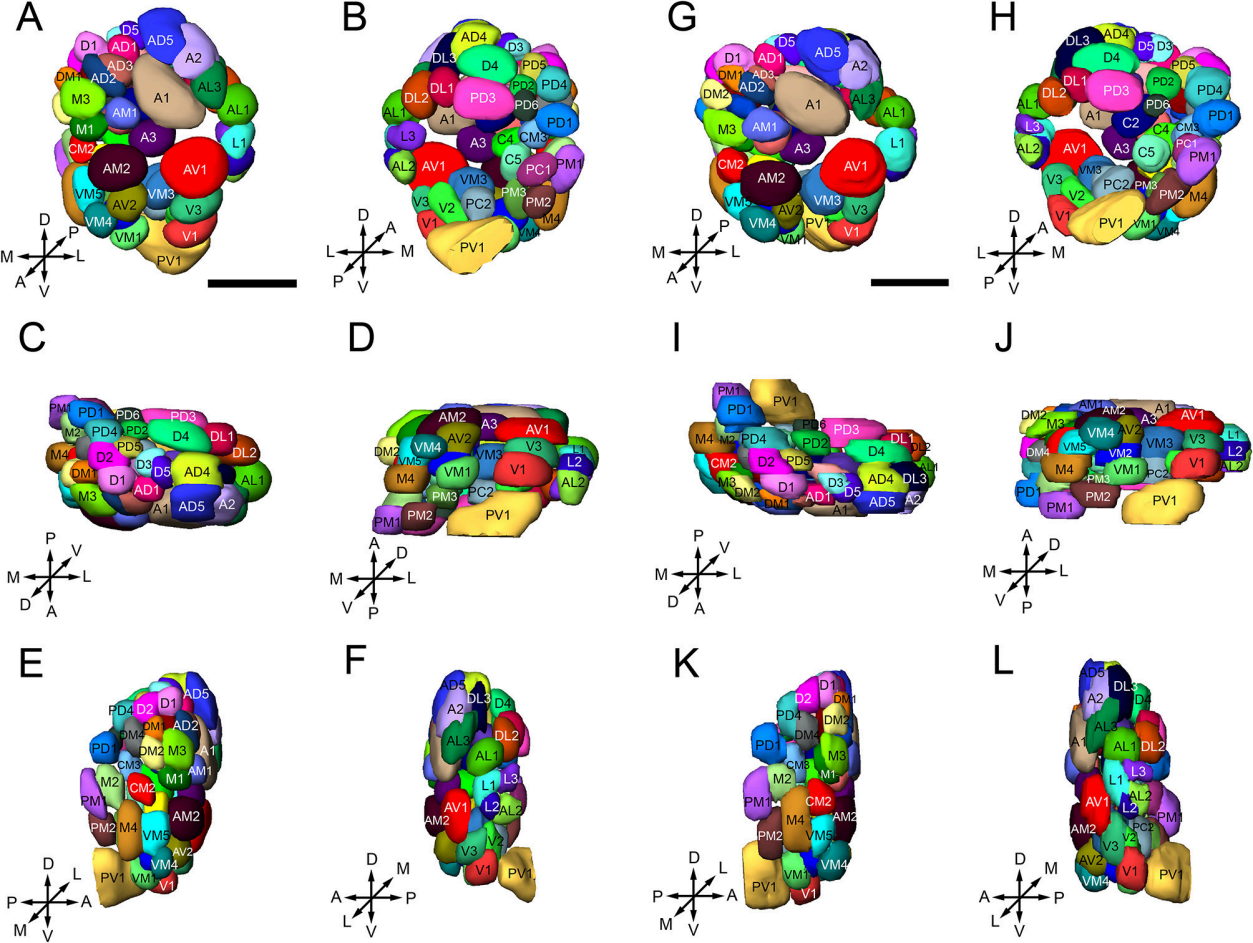
Three-dimensional reconstruction of the glomeruli in the right AL of *B. dorsalis* [**(A–F)** is male, **(G–L)** is female], shown in the anterior **(A,G)**, posterior **(B,H)**, dorsal **(C,I)**, ventral **(D,J)**, medial **(E,K)**, and lateral**(F,L)** part of the AL. Glomeruli are visualized with different colors to distinguish single units, and named according to Solari et al. (2016) and Stocker (1994). A total of 64 or 65 glomeruli can be identified in both sexes. The orientation of the AL is indicated by the crosses: A, anterior; P, posterior; D dorsal; V, ventral; L, lateral; M, medial. Scale bars = 50 μm.

The landmark glomerulus A3 was easily identifiable by its size and position (Figures 2B,C, 4A,G), which led to identification of AM2 and AV2 on the posterior side of the A3. Dorsomedial to the A3 are the A1 and AM1 (Figures 2B, 4A,G). Adjacent to the A1, from ventral to dorsal side, the glomeruli AD1-3, AD5, A2, and AL3 can be located (Figures 2C, 4A,G).

#### Dorsal region

D1, D2, and D4 are the most conspicuous glomeruli in the dorsal region (Figures 2D–G, 4C,I). From medial to lateral side, D3, D5, AD4, and DL3 can be found. AD4 is located posteriorly to AD5. Surrounding the D4 is a group of glomeruli including PD2, PD3, PD5, PD6, and DL1 (Figures 2F–I, 4B,H). PD2 is located on the medial side of D4, PD3 is located on the posterior side, PD5 is located on the anterior medial side, PD6 is located on the medial-posterior side, and DL1 is located on the lateral-posterior side. Adjacent to the D2 and PD5, the glomeruli PD4 and PD1 are located. PD4 is on the lateral and ventral posterior side. Close to the D1 and D2, from dorsal to ventral side, the glomeruli DM1-2, DM4, M3, and M1 are identified. DM3 is situated at the ventral side of D1.

#### Ventral region

V1 is easily identifiable from its shape and position (Figures 2F,G, 4D,J). Starting from V1, we can find a group of glomeruli that include V2-3, VM1-3, and PC2 (Figures 2E–G, 4D,J). V2 is located laterally and dorsoposteriorly to the V1, V3 is located on the anterior-lateral side, VM1 is located on the medial side. VM2 is positioned medially and dorsoanteriorly to the VM1. VM3 is located medial-dorsally to the V2. One large glomerulus, PV1, is located most posteriorly in the ventral part of the AL (Figures 2H–J, 4D,J).

#### Lateral region

Glomeruli at the lateral region of the AL could be identified based on their positions relative to the antennal mechanosensory and motor center (AMMC) (Figures 3C,D). This group comprises DL2, AL1, L1-3, and AL2 (Figures 2E–H, 4F,L). DL2 is positioned at the lateral side of the DL1, dorsal to AL1. L1 and L2 is located ventrally to the AL1, L3 is on the posterior side of L1. AL2 is located posteriorly to the L2.

#### Central region

The landmark glomeruli C3, C4, and CM2 were easily identifiable by their size and positions (Figures 2E,F). According to these landmarks, the glomeruli C1-2, C5, DC1-3, CM1, and CM3 are identified (Figures 2D–F). Among them, C3, C4, C2, and DC2 are arranged in sequence, DC1 and DC3 are located laterally and dorsally to the DC2 and C2, respectively (Figures 2E,F). CM1 is located posteriorly to C4, and CM3 is located medially and dorsoanteriorly to the C4. C5 is on the ventral side of the CM3.

#### Medial region

When we examined the medial part glomeruli of the AL, we found that some glomeruli were not labeled by the Micro-Ruby fluorescent dye that was applied anterogradely from the antennal OSNs (Figures 3B–F), but was clearly labeled by anterograde staining from the maxillary palp nerve (Figures 3I–K). The stained axon bundle of the sensory neurons entered the ipsilateral side of the gnathal ganglia (GNG) via the maxillary palp nerve, terminating in a set of medial (M) part glomeruli in both the ipsi- and contra-lateral AL with the contralateral afferents crossing the midline via the inferior AL commissure (iALC) (Figure 3G). We identified 9 glomeruli innervated by the maxillary palp nerve, including VM4, VM5, M2, M4, CM3, PM1, PM2, PM3, and PC1 (Figures 3H–K). Adjacent to the CM3, from dorsal to ventral side, the glomeruli M2, M4, PM1, and PC1 are located (Figures 4E,K). M2 is situated ventromedially to the CM3, whereas M4 and PM1 are positioned medioanteriorly and medioposteriorly to the CM3, respectively. PC1 is located ventrally and dorsoposteriorly to the CM3. PM2 is located on the ventroposterior side of the M4, and PM2 is on the posterior side of the PM3. Two easily recognizable glomeruli located adjacent to M4 are VM5 and VM4. VM5 is on the anterior side of the M4, whereas VM4 is on the ventral side of the VM5.

### Glomerular volume of males and females

**Figure 5:**
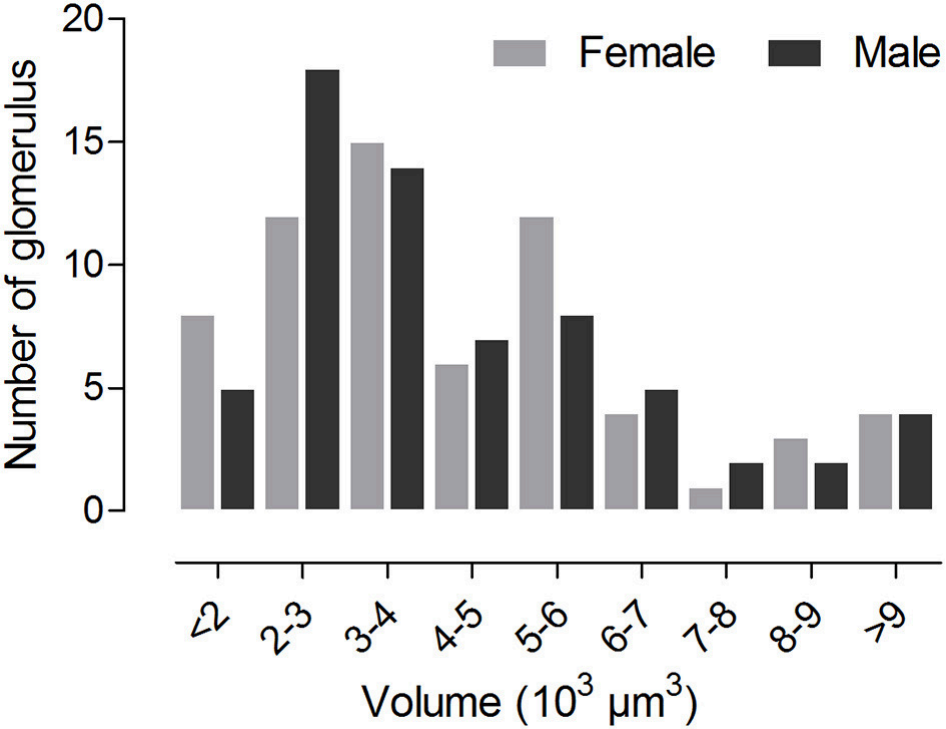
Histogram showing number of glomeruli possessing distinct volume.

The volume of the individual glomeruli varies from 897 to 19,592 μm^3^ in females, and from 806 to 18,294 μm^3^ in males, respectively (Table 1). The largest glomerulus in both the female and male AL is the PV1, with mean volume of 19,279 and 16,663 μm^3^, which accounts for 6.39 and 5.66% of the total glomerular volume. Most of the glomeruli, about 47 in male, 44 in male, shows volume between 2,000 and 6,000 μm^3^ (Figure 5, **Table 3**), and accounts for 58.8 and 57.1% of the total glomerular volume, respectively. Four glomeruli, including AM2, AV1, A1, PV1, have volume larger than 9,000 μm^3^ in both female and male (Figure 5, **Table 3**). The 9 glomeruli innervated by the maxillary palp nerve, constitutes 13.2 and 13.4% in female and male, of the total volume of all glomeruli. Among glomeruli innervated by the maxillary palp, M4 is larger than others, and accounts for 22.56 and 20.66% of these nine glomeruli in female and male respectively.

### Comparison of male and female ALs

The three-dimensional atlas using the same naming and color-coding system for glomeruli facilitates comparisons between the ALs of the female and male (Figure 4, Supplementary Presentation 1). We compared the size of the 65 homologous (isomorphic and sexually dimorphic) glomeruli in males and females by using correlation coefficients (Table 2). Generally, the correlations in corresponding glomeruli between the right and left ALs of the same brain and across male and female individuals are above 0.8, indicating that the homologous glomeruli have similar size in the left and right AL, and this phenomenon is also true in different individuals in both sexes (Table 2).

**Table 2:** Coefficient of correlation (*r*) of glomerular size within, between individuals and sexes.

**Comparison**	**Comparison pairs**	**Number of glomeruli paired**	**Size *r***	***P*-value**
Intra-individual Female	Female1 R/L	64	0.956	<0.001
	Female2 R/L	65	0.845	<0.001
	Female3 R/L	64	0.932	<0.001
Inter-individual Female	Female1/Female2	64	0.909	<0.001
	Female1/Female3	64	0.893	<0.001
	Female2/Female3	64	0.889	<0.001
Intra-individual male	Male1 R/L	65	0.843	<0.001
	Male2 R/L	64	0.951	<0.001
	Male3 R/L	64	0.897	<0.001
Inter-individual male	Male1/Male2	64	0.892	<0.001
	Male1/Male3	64	0.881	<0.001
	Male2/Male3	64	0.875	<0.001
Inter-sexual	M1/F1	63	0.907	<0.001
	M1/F2	65	0.910	<0.001
	M1/F3	64	0.892	<0.001
	M2/F1	63	0.863	<0.001
	M2/F2	64	0.864	<0.001
	M2/F3	63	0.829	<0.001
	M3/F1	63	0.868	<0.001
	M3/F2	64	0.899	<0.001
	M3/F3	63	0.886	<0.001

*Male1-Male3 indicate three male individuals. Female1-Female3 indicate three female individuals. R and L indicate the right and left antennal lobe, respectively*.

**Figure 6:**
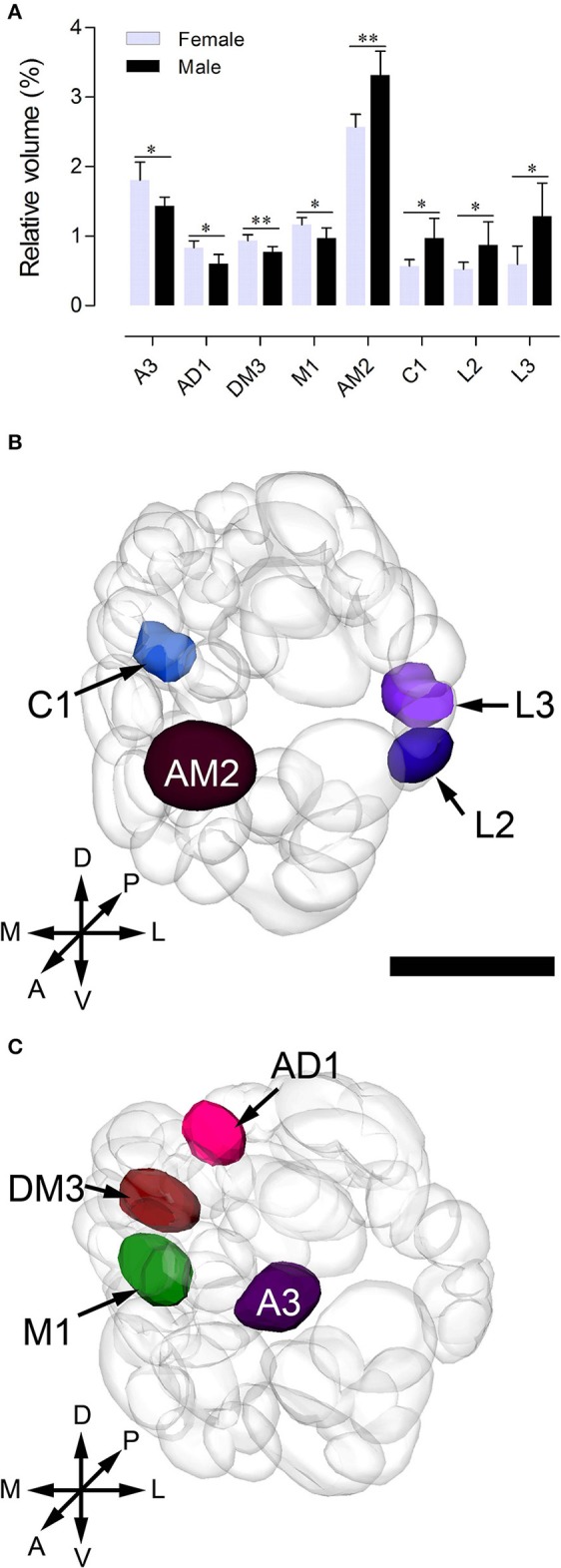
Sexually dimorphic glomeruli in *B. doraslis*. **(A)** Eight glomeruli show significant differences between females and males (*P < *0.05;* **P < *0.01*; *Mann-Whitney U*-test); four are larger in males, and four are larger in females. **(B–C)** Three-dimensional reconstructions of the ALs indicating the location of the enlarged glomeruli in male **(B)** and female **(C)** from the anterior view. A, anterior; P, posterior; D, dorsal; V, ventral; L, lateral; M, medial. Scale bars = 50 μm in **(B)** and applies to **(C)**.

Sexual variation of glomerular organization was also examined in 12 ALs (from 3 males and 3 females). The non-parametric *Mann-Whitney U*-test indicated no significant difference in the total glomerular volume in left and right AL for both sexes, and the same is true when comparison was made between sexes (Table 3). The mean total volume is 301,550 μm^3^ in females and 294,229 μm^3^ in males (*P* = 0.394) (Table 3). However, we did find sexual dimorphism in some glomeruli. Four glomeruli are larger in males: AM2 (*P* = 0.002), C1 (*P* = 0.015), L2 (*P* = 0.015), and L3 (*P* = 0.026) (Table 3, Figure 6A). The volume of AM2 is 9,762 μm^3^ (*n* = 6), much larger than L2 and L3, and this glomerulus is located medially in the anterior region (Figure 6B). C1 is located in the central region, L2 and L3 are located in the lateral part of the AL. Four glomeruli are larger in females: A3 (*P* = 0.026), M1 (*P* = 0.041), DM3 (*P* = 0.009), and AD1 (*P* = 0.015) (Table 3, Figure 6A). In these four glomeruli, A3 is the largest with total volume of 5,443 μm^3^ (*n* = 6, Figure 6A). This glomerulus is located in the anterior region (Figure 6C). The volume of remaining three glomeruli are smaller than A3. DM3 and M1 are located in the medial part of the dorsal region, whereas AD1 is dorsally situated in the anterior region (Figure 6C).

**Table 3:** Quantitative comparison of individual glomeruli of female and male AL.

**Glomeruli**	**Female**		***n***	**Male**		***n***	***U*-test for right and left AL of female**	***U*-test for right and left AL of male**	***U*-test for female and male**
	**Volume (10^3^ μm^3^)**	**Relative volume (%)**		**Volume (10^3^ μm^3^)**	**Relative volume (%)**		***P*-value**	***P*-value**	***P*-value**
A1	13.23 ± 1.94	4.39 ± 0.52	6	12.58 ± 1.16	4.28 ± 0.50	6	0.100	1.000	0.589
A2	5.99 ± 2.28	1.99 ± 0.71	6	6.44 ± 1.47	2.19 ± 0.48	6	0.700	0.700	0.485
A3	5.44 ± 0.83	1.81 ± 0.26	6	4.22 ± 0.32	1.43 ± 0.13	6	0.700	0.700	0.026*
AD1	2.50 ± 0.21	0.83 ± 0.10	6	1.77 ± 0.38	0.60 ± 0.14	6	0.700	1.000	0.015*
AD2	2.25 ± 0.49	0.75 ± 0.16	6	1.61 ± 0.53	0.55 ± 0.19	6	1.000	1.000	0.180
AD3	1.58 ± 0.49	0.54 ± 0.14	6	1.87 ± 0.50	0.64 ± 0.18	6	0.400	0.700	0.240
AD4	5.34 ± 1.74	1.77 ± 0.59	6	6.97 ± 1.61	2.37 ± 0.52	6	0.700	0.700	0.132
AD5	5.15 ± 1.02	1.71 ± 0.36	6	6.40 ± 1.43	2.17 ± 0.48	6	1.000	0.700	0.065
AL1	5.51 ± 0.93	1.83 ± 0.24	6	4.29 ± 1.64	1.46 ± 0.59	6	0.700	1.000	0.485
AL2	3.12 ± 1.60	1.03 ± 0.57	6	2.99 ± 1.60	1.02 ± 0.56	6	1.000	1.000	1.000
AL3	6.11 ± 1.80	2.03 ± 0.57	6	5.30 ± 2.60	1.80 ± 0.91	6	0.400	0.700	0.485
AM1	2.73 ± 0.93	0.91 ± 0.29	6	2.47 ± 0.94	0.84 ± 0.36	6	1.000	0.100	0.937
AM2	7.74 ± 0.78	2.57 ± 0.19	6	9.76 ± 1.12	3.32 ± 0.34	6	0.100	0.400	0.002*
AV1	3.87 ± 0.92	1.28 ± 0.27	6	4.40 ± 0.50	1.49 ± 0.19	6	0.700	0.700	0.093
AV2	10.82 ± 1.41	3.59 ± 0.45	6	11.31 ± 1.42	3.84 ± 0.39	6	1.000	0.700	0.485
C1	1.71 ± 0.27	0.57 ± 0.09	6	2.85 ± 0.85	0.97 ± 0.29	6	1.000	1.000	0.015*
C2	5.29 ± 1.37	1.75 ± 0.50	6	5.98 ± 1.22	2.03 ± 0.37	6	0.700	0.700	0.589
C3	3.39 ± 0.84	1.12 ± 0.31	6	3.74 ± 1.40	1.27 ± 0.46	6	1.000	1.000	0.699
C4	4.82 ± 0.65	1.60 ± 0.16	6	4.86 ± 0.89	1.65 ± 0.28	6	0.700	0.200	0.699
C5	3.53 ± 0.63	1.17 ± 0.18	6	3.80 ± 1.07	1.29 ± 0.34	6	1.000	0.200	0.589
CM1	2.84 ± 1.41	0.94 ± 0.44	6	2.65 ± 0.60	0.90 ± 0.19	6	0.400	0.700	1.000
CM2	3.45 ± 0.65	1.14 ± 0.19	6	2.72 ± 0.34	0.93 ± 0.12	6	1.000	1.000	0.093
CM3	2.71 ± 0.58	0.90 ± 0.20	6	2.95 ± 0.65	1.00 ± 0.22	6	0.400	0.700	0.310
D1	3.57 ± 0.70	1.18 ± 0.23	6	3.13 ± 0.60	1.06 ± 0.20	6	1.000	0.100	0.485
D2	3.71 ± 0.30	1.23 ± 0.08	6	3.63 ± 0.64	1.23 ± 0.20	6	1.000	0.400	0.589
D3	1.53 ± 0.29	0.51 ± 0.08	6	1.70 ± 0.53	0.58 ± 0.17	6	0.700	0.700	0.589
D4	8.04 ± 1.42	2.67 ± 0.44	6	7.01 ± 0.53	2.38 ± 0.16	6	1.000	1.000	0.485
D5	2.76 ± 0.41	0.92 ± 0.15	6	2.39 ± 0.84	0.81 ± 0.29	6	0.700	1.000	0.394
DC1	3.48 ± 0.85	1.15 ± 0.26	6	2.50 ± 1.01	0.85 ± 0.35	6	0.400	0.700	0.132
DC2	6.43 ± 0.96	2.13 ± 0.40	6	5.07 ± 1.79	1.72 ± 0.56	6	0.700	1.000	0.240
DC3	1.45 ± 0.21	0.48 ± 0.24	2	2.72 ± 1.51	0.92 ± 0.51	6	–	1.000	–
DL1	3.74 ± 0.63	1.24 ± 0.22	6	3.95 ± 0.23	1.34 ± 0.09	6	0.700	0.700	0.589
DL2	4.91 ± 1.24	1.63 ± 0.39	6	4.57 ± 1.20	1.55 ± 0.44	6	1.000	1.000	0.937
DL3	5.94 ± 1.4	1.97 ± 0.47	6	5.55 ± 1.48	1.89 ± 0.49	6	0.400	1.000	0.589
DM1	1.70 ± 0.30	0.56 ± 0.10	6	2.07 ± 0.45	0.70 ± 0.16	6	1.000	0.700	0.180
DM2	2.72 ± 0.60	0.90 ± 0.22	6	2.24 ± 0.74	0.76 ± 0.27	6	1.000	0.200	0.394
DM3	2.82 ± 0.34	0.94 ± 0.08	6	2.27 ± 0.15	0.77 ± 0.08	6	1.000	0.100	0.009*
DM4	4.09 ± 0.74	1.36 ± 0.21	6	3.51 ± 0.18	1.19 ± 0.09	6	1.000	0.400	0.093
L1	2.89 ± 0.98	0.96 ± 0.33	6	2.87 ± 0.78	0.98 ± 0.26	6	0.700	0.400	0.937
L2	1.58 ± 0.29	0.53 ± 0.10	6	2.57 ± 1.00	0.87 ± 0.33	6	0.700	1.000	0.015*
L3	1.79 ± 0.77	0.59 ± 0.26	6	3.79 ± 1.47	1.29 ± 0.47	6	0.700	0.400	0.026*
M1	3.51 ± 0.32	1.16 ± 0.10	6	2.85 ± 0.42	0.97 ± 0.15	6	1.000	1.000	0.041*
M2	4.86 ± 2.54	1.61 ± 0.78	6	5.65 ± 1.63	1.92 ± 0.50	6	0.700	0.700	0.240
M3	3.88 ± 1.39	1.29 ± 0.41	6	3.57 ± 1.42	1.22 ± 0.50	6	0.400	0.400	1.000
M4	9.15 ± 1.16	3.03 ± 0.48	6	8.02 ± 1.03	2.73 ± 0.39	6	1.000	1.000	0.240
PC1	2.09 ± 0.55	0.69 ± 0.21	6	3.58 ± 2.09	1.22 ± 0.67	6	0.700	1.000	0.180
PC2	3.46 ± 1.95	1.15 ± 0.63	6	3.00 ± 0.86	1.02 ± 0.27	6	1.000	1.000	0.937
PD1	6.88 ± 0.93	2.28 ± 0.30	6	5.59 ± 1.19	1.90 ± 0.37	6	1.000	1.000	0.093
PD2	2.65 ± 0.64	0.88 ± 0.19	6	2.67 ± 0.71	0.91 ± 0.22	6	0.200	0.400	0.937
PD3	8.59 ± 1.13	2.85 ± 0.35	6	7.73 ± 0.91	2.63 ± 0.26	6	1.000	0.700	0.310
PD4	5.46 ± 1.02	1.81 ± 0.28	6	4.70 ± 0.82	1.60 ± 0.24	6	1.000	0.400	0.485
PD5	2.18 ± 0.43	0.72 ± 0.16	6	2.01 ± 0.16	0.68 ± 0.06	6	0.700	0.400	0.310
PD6	1.5 ± 0.34	0.50 ± 0.12	6	1.25 ± 0.26	0.43 ± 0.24	4	1.000	1.000	0.132
PM1	5.53 ± 2.57	1.84 ± 0.90	6	3.19 ± 1.23	1.08 ± 0.42	6	1.000	1.000	0.132
PM2	3.87 ± 1.04	1.28 ± 0.36	6	3.85 ± 1.10	1.31 ± 0.39	6	1.000	1.000	0.699
PM3	3.44 ± 0.97	1.14 ± 0.31	6	3.15 ± 0.75	1.07 ± 0.26	6	1.000	0.700	0.937
PV1	19.28 ± 3.23	6.39 ± 1.08	6	16.66 ± 1.45	5.66 ± 0.41	6	0.100	0.400	0.240
V1	6.80 ± 0.80	2.26 ± 0.30	6	6.34 ± 0.70	2.16 ± 0.23	6	1.000	0.700	0.589
V2	3.24 ± 0.55	1.08 ± 0.19	6	3.92 ± 0.73	1.33 ± 0.23	6	0.400	0.700	0.093
V3	5.14 ± 0.50	1.71 ± 0.14	6	5.65 ± 0.68	1.92 ± 0.22	6	0.700	0.400	0.065
VM1	5.78 ± 1.48	1.92 ± 0.50	6	6.10 ± 3.33	2.07 ± 1.24	6	0.400	1.000	0.485
VM2	5.36 ± 0.63	1.78 ± 0.19	6	4.50 ± 0.67	1.53 ± 0.21	6	0.700	0.200	0.093
VM3	8.69 ± 0.74	2.88 ± 0.15	6	8.79 ± 1.14	2.99 ± 0.36	6	0.700	0.400	0.485
VM4	4.05 ± 1.18	1.34 ± 0.36	6	3.16 ± 0.64	1.08 ± 0.18	6	0.400	0.400	0.240
VM5	4.86 ± 0.44	1.61 ± 0.18	6	5.28 ± 0.75	1.80 ± 0.23	6	0.400	0.400	0.180
Sum of All glomeruli	301.55 ± 13.78	_	6	294.23 ± 10.06	_	6	0.200	0.400	0.394

*Volume and relative volume are presented as Mean ± SD. Relative volume was used for statistical comparison in order to control for the variation in absolute volume in individuals, except for comparing the total volume of all glomeruli. Asterisks indicate statistical significance at the level of P = 0.05*.

### Afferent innervation of AL glomeruli

Anterograde staining of the antennal and maxillary palps OSNs revealed their innervation pattern in the glomerular array. In our observation, 56 glomeruli are found to be innervated by antenna afferents (Figures 3A–F), and 9 medial glomeruli only receive maxillary palp afferents (Figures 3H–I). It appears that these two sensory organs target different subsets of glomeruli.

**Figure 7:**
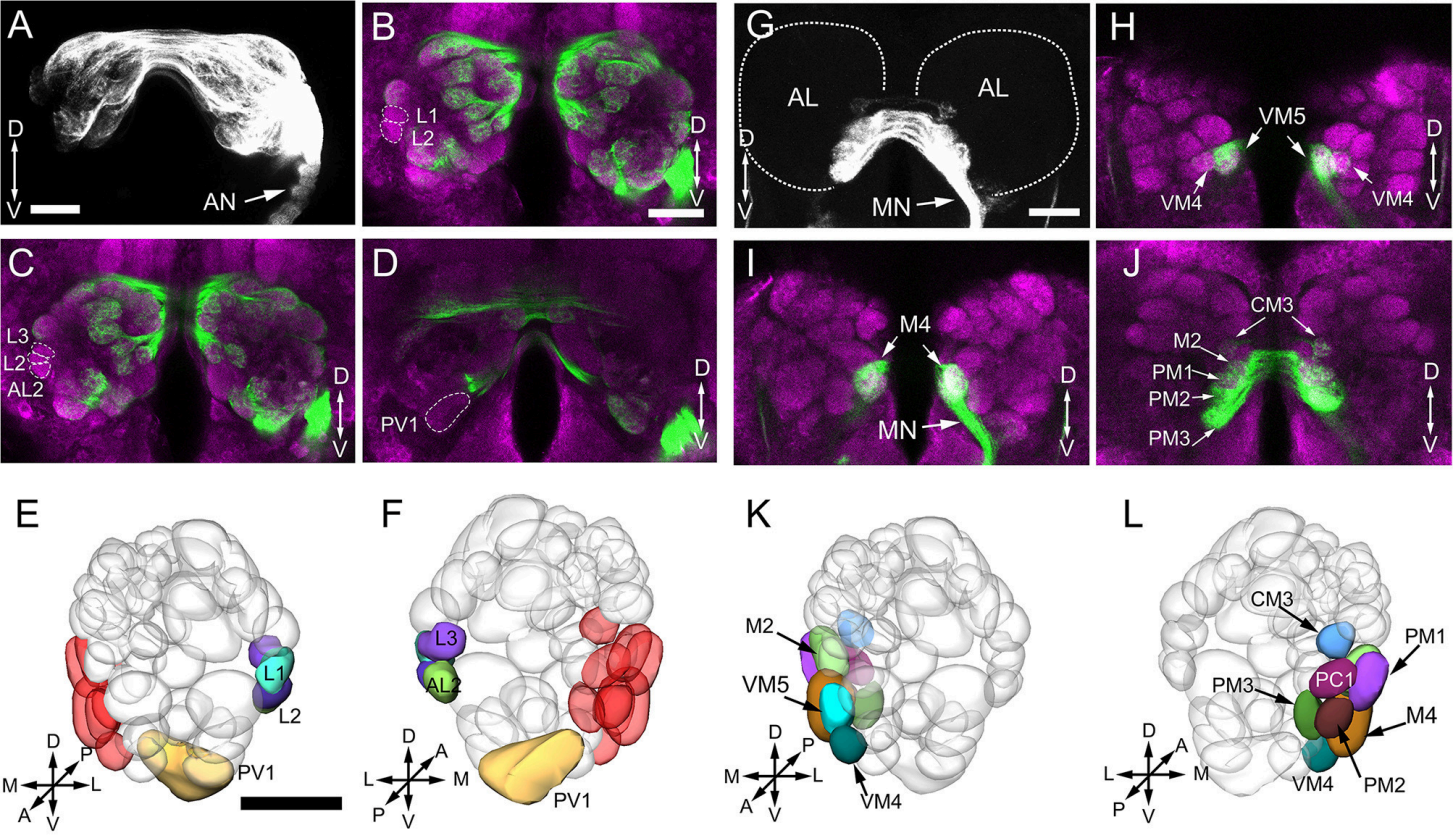
Afferent innervation of AL glomeruli. **(A)** Projected view of AL innervated by antennal ascending neurons. Backfills with TMR from the unilateral (right) antennal show that not only the ipsilateral glomeruli are stained, but also the contralateral glomeruli. **(B–D)** Confocal sections in different planes showing five glomeruli free of staining by contralateral antennal nerves (green); brain structures are marked by means of synapsin immunolabeling (magenta). **(E–F)** Three-dimensional reconstructions of the ALs showing the glomeruli innervated by ipsilateral antenna ascending neurons from anterior **(E)** and posterior **(F)** view. The medially located glomeruli in red color are not labeled by antenna backfill. **(G)** Projected views of glomeruli innervated by maxillary papls sensory neurons. Backfills with TMR from the unilateral (right) labeled glomeruli bilaterally. **(H–J)** Confocal sections in different planes showing the right and left glomeruli in AL staining by the unilateral maxillary palp nerves. **(K,L)** Three—dimensional reconstructions of the ALs indicating 9 glomeruli innervated by maxillary palp sensory neurons from anterior **(K)** and posterior **(L)** view. AN, antennal nerve; MN, maxillary palp nerve; OE, esophagus; A, anterior; P, posterior; D, dorsal; V, ventral; L, lateral; M, medial. Scale bars 100 μm in **(A,B,G)** (applies to **B–D, H–J)**, 50 μm in E (applies to **F**,**K**,**L**).

In this study, we also found some interesting projection patterns. First, some antennal afferents project to the ipsilateral AL, and then through the antennal lobe commissure (ALC) to the homologous glomeruli on the contralateral side (Figure 7A). Five glomeruli, named L1, L2, L3, AL2, and PV1 are exceptional; they only receive ipsilateral fibers (Figures 7B–D, show the location in Figures 7E,F). Second, afferents from the maxillary palps are all bilateral fibers (Figure 7G), i.e., they innervate the ipsilateral AL, and then through the inferior antennal lobe commissure (iALC) extend to the homologous glomeruli on the contralateral side (Figures 7H–J, location shown in Figures 7K,L).

### Antennal lobe tracts

**Figure 8:**
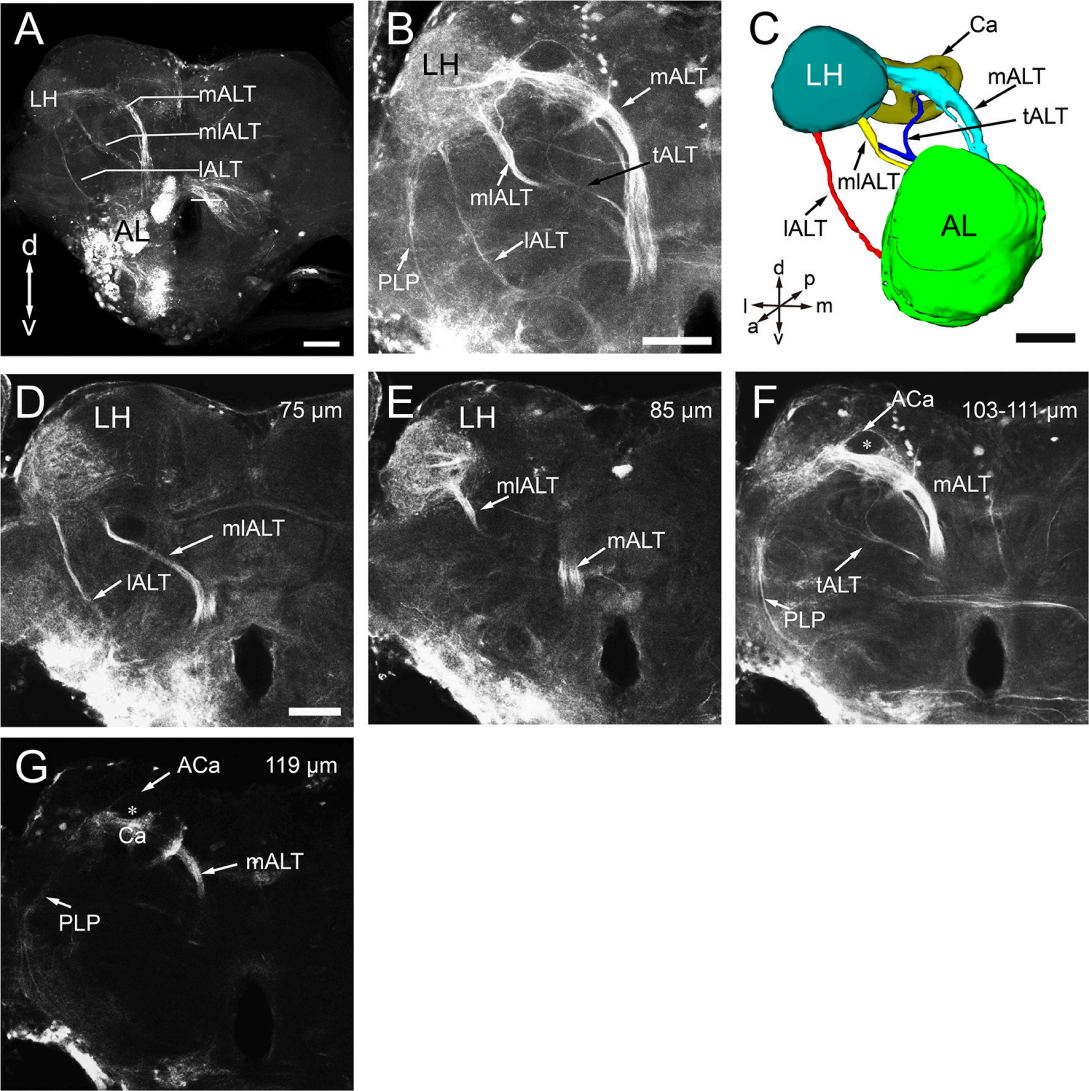
Antennal lobe tracts (ALTs) in the left hemisphere of *B. dorsalis*. **(A)** Projected views of the three main ALTs, the medial ALT (mALT), the medio-lateral ALT (mlALT), and the lateral ALT (lALT). **(B)** Projected view of one brain showing the transverse ALT (tALT) (black arrow). **(C)** Three-dimensional reconstructions of the four ALTs in anterior view. **(D–G)** Confocal sections of the ALTs in different planes, obtained by applying dye into the antennal lobe (same confocal stack as **B)**. The prominent mALT projects to the calyces (Ca) before terminating in the lateral horn (LH) **(F,G)**, whereas the mlALT and the lALT directly project to the LH **(D,E)**. However, the lALT and mlALT project into different zones of the LH with the lALT terminating in the lateral part and mlALT in medial LH **(D,E)**. Moreover, several fiber bundles from the LH target the posterior lateral protocerebrum (PLP) (**B**,**F**,**G**). From anterograde staining in the AL, only the calyx can be stained in the mushroom body, accessory calyx (ACa) are free of staining (the star in **F**,**G)**. A, anterior; P, posterior; D, dorsal; V, ventral; L, lateral; M, medial. Scale bars 100 μm in **(A)**, 50 μm in **(B**,**C)** (applies to **E–G)**.

Like in other insects, several ALTs in the oriental fruit fly brain connect the AL to higher processing centers, including the calyces of mushroom bodies (MBs) and the lateral horn (LH) (Homberg et al., 1988; Rø et al., 2007). Following the established nomenclature (Ito et al., 2014), these tracts are called medial, mediolateral, and lateral antennal-lobe tracts, i.e., mALT, mlALT, and lALT (Figure 8A). The mlALT, and lALT appear to be thinner than the mALT, and they project directly to the medial and lateral region of the LH, respectively (Figures 8B–E). The mALT first projects to the ipsilateral calyx (Ca), and then terminates at the LH (Figures 8B,C). Neither the mALT nor the lALT projects to the accessory calyx (ACa) (Figures 8F,G), which most likely receives visual input (Vogt et al., 2016). Another less commonly seen tract, namely transverse ALT or tALT, following a report on *D. melanogaster* (Tanaka et al., 2012b), is also present in our preparation. This tract exits AL with the mALT for a short distance, and then turns laterally before splitting to two branches. The first branch targets the ipsilateral calyx (Figures 8C,F), and second branch projects slightly anteriorly and joins the mlALT before ending in the LH (Figure 8C).

### Anatomical organization of medial tract neurons in the AL

**Figure 9:**
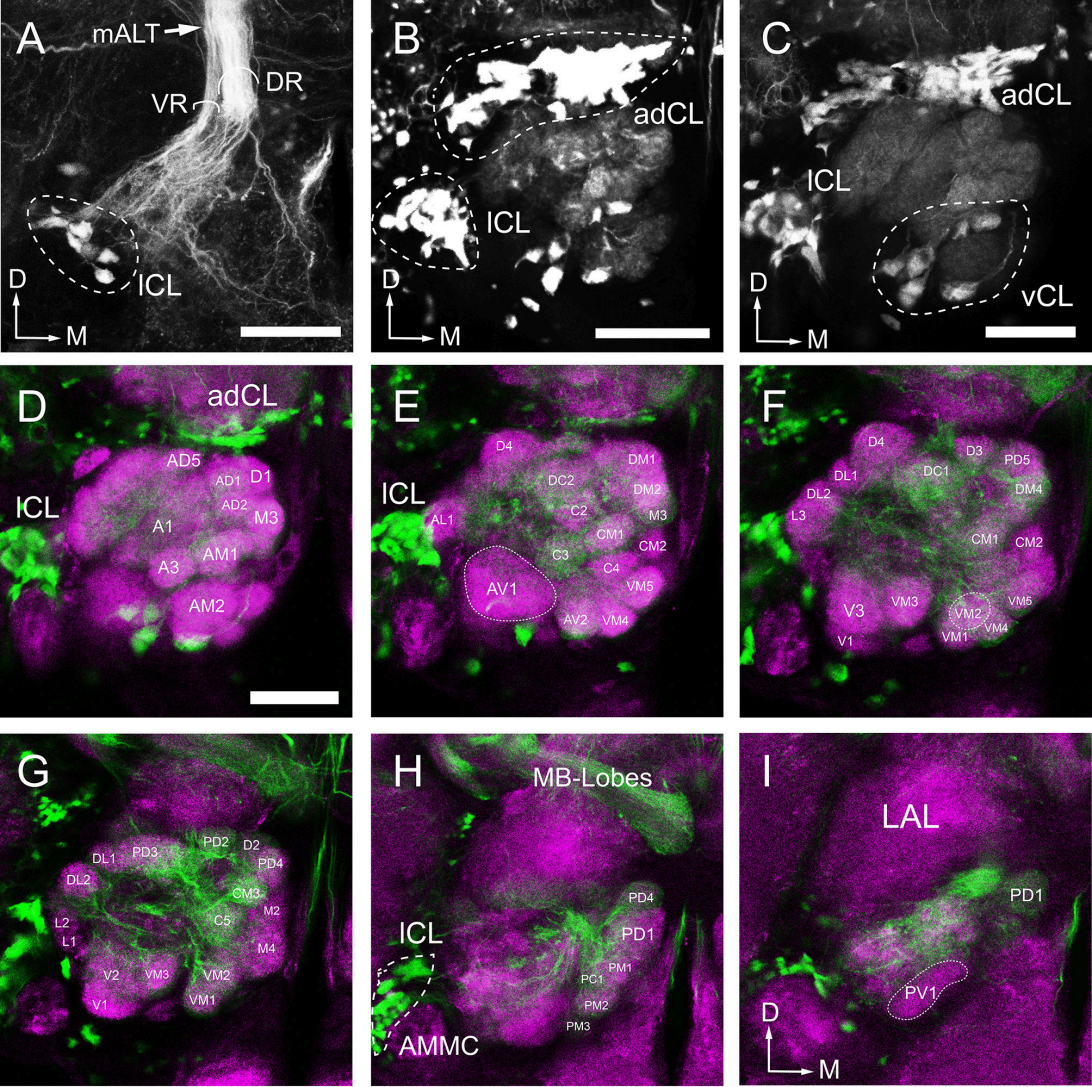
Arborization pattern and cell body clusters of mALT neurons in the AL, visualized by applying dye in the calyx. **(A,B)** The mALT divides into two fiber bundles before reaching into the AL: the dorsal root (DR) and the ventral root (VR) (named follow Ian et al., 2016) **(A)**. The DR is connected to the anterodorsal cluster (adCL) and the lateral (lCL) of cell bodies **(B)**. The cell body cluster of the VR neurons is located more ventroposteriorly, separated from the DR cell body clusters. **(C)** A small group of cell bodiesis located ventrally to the AL (vCL), and they send a very thin fibers projecting along the peripheral part of the AL. **(D–I)** Confocal sections showing the glomeruli innervated by mALT neurons. The mALT (green) is visualized via retrograde mass staining in the calyces. Central brain is marked by means of synapsin immunolabeling (magenta). Most glomeruli, but not all, are innervated by the stained mALT. From anterior to posterior, the labeled glomeruli are shown at depth of 14 μm **(D)**, 28 μm **(E)**, 36 μm **(F)**, 42 μm **(G)**, 48 μm **(H)**, and 56 μm **(I)**. LAL, Lateral accessory lobe; AMMC, antennal mechanosensory and motor center; MB-Lobes, mushroom body lobes; D, dorsal; M, medial. Scale bar = 50 μm in **(A**–**D)** (applies to **E**–**I)**.

To further characterize the glomerular output to the MB calyx (Ca), we applied retrograde staining from Ca. Results show that, among the ALTs, the mALT is the only tract supplying the Ca, and it is consisted of two roots: the thicker dorsal root (DR) and the thinner ventral root (VR) (Figure 9A). The somata of the PNs whose axons form the DR are situated in two clusters: the anterodorsal cluster (adCL) and lateral cluster (lCL) (Figure 9B). adCL seems larger than the lCL. The VR PNs have their somata located in the lCL as well (Figure 9A), but occupying the ventroposterior area of the lCL, dorsal to the antenna mechanic motor center (AMMC) (Figures 9A,H). In addition to the two roots, a few scattered branches extending from the mALT are linked to the ventral cluster (vCL), where approximately 8 cell bodies can be counted (Figure 9C). The mALT PNs innervate most glomeruli, except for PV1, AV1, L1, L2, L3, and AL2 (Figures 9D–I).

## Discussion

### Number of AL glomeruli

Generally, the number of glomeruli is species specific. For example, most species of moths and butterflies have 60–70 glomeruli (Greiner et al., 2004; Couton et al., 2009; Varela et al., 2009; Løfaldli et al., 2010; Heinze and Reppert, 2012; Montgomery and Ott, 2015); different species of bees have 100–210 (Streinzer et al., 2013; Roselino et al., 2015); Parasitoid wasps have approximately 200 (Smid et al., 2003); Ants have 100–600 (Kelber et al., 2009; Mysore et al., 2009; Nakanishi et al., 2010; Stieb et al., 2011); Locusts have more than 1,000 so called microglomeruli (Ignell et al., 2001). Overall, most insects have 50–200 glomeruli (Anton and Homberg, 1999; Schachtner et al., 2005).

We describe, for the first time, 64–65 identifiable glomeruli in both sexes in *B. dorsalis*. This number is within the range of 50–70 for Dipterans (Laissue et al., 1999; Ignell et al., 2005; Ghaninia et al., 2007; Riabinina et al., 2016). A tephritid fly, *Ceratitis capitata*, which is phylogenetically close to *B. dorsalis*, was reported to have only 53 glomeruli (Solari et al., 2016). However, in that study, the authors counted only the glomeruli that were stained by TMR anterograde backfills from the antennal OSNs, whereas the glomeruli innervated by the maxillary-palp nerves were overlooked. In our study, nine glomeruli located in the medial region receiving inputs from the maxillary palp afferents were identified (Figures 3, 7). The number of glomeruli innervated by antennal OSNs in *B. dorsalis* is slightly higher than that in *C. capitate*, 56 vs. 53, respectively. This small discrepancy may be due to the use of slightly different criteria for judging glomerular boundaries between the two studies. Similar phenomena were also reported in other studies (Couton et al., 2009). Typically, one type of olfactory receptor neuron (ORNs) expresses a given odorant receptor and one glomerulus receives OSNs expressing a given odorant receptor, following the “one receptor—one ORN—one glomerulus” principle, except for a few examples (Hansson et al., 1996; Goldman et al., 2005; Hansson and Stensmyr, 2011). The antennal transcriptome analyses of *B. dorsalis* resulted in identification of a total of 43 odorant receptors (ORs), 12 gustatory receptors (GRs), and 21 ionotropic receptors (IRs) (Wu Z. et al., 2015; Liu et al., 2016; Jin et al., 2017), which roughly correspond to the number of glomeruli innervated by the antennal afferents.

Two particular glomeruli, PD6 in male and DC3 in female, are occasionally missing, resulting in total counts of 64 or 65. Such marginal differences were also observed in other insects (Rospars and Hildebrand, 1992, 2000; Galizia et al., 1999; Couton et al., 2009; Kazawa et al., 2009). This may be associated with poor penetration of the synapsin antibody in the deeper brain tissue, making it difficult to ascertain the border of these two glomeruli. Nonetheless, we still cannot eliminate the possibility of variable number of glomeruli across individuals (Rospars and Hildebrand, 2000; Couton et al., 2009), which may reflect genetic variability of the olfactory receptors.

### Sexual dimorphism in the AL

By comparing the reconstructed atlases of the ALs, we found that some glomeruli, which occupy similar positions in both sexes, were enlarged in females or males. However, we did not observe anything resembling the macroglomerular complex (MGC) in moths. In our study, eight glomeruli are sexually dimorphic, four of which, AM2, C1, L2, and L3, are larger in males, and four of which, A3, AD1, DM3, and M1, are larger in females (Figure 6). This phenomenon also occurs in other Dipteran insects. Kondoh et al. (2003) compared the AL of 37 species of *Drosophilidae* from the islands of the Hawaiian archipelago, and found that two glomeruli DA1 and DL3, are markedly larger in males in six species. Additionally, in *Aedes aegypti*, the glomeruli AM1-2, AD1-2, PM1-3, V1-4 are sexually dimorphic in size and shape (Ignell et al., 2005). In the family of tephritidae, *C. capitata* females have three (named PD1, C3, and PV1) enlarged glomeruli, while males have four enlarged glomeruli (Solari et al., 2016). In some species, sexual dimorphism in glomerular organization indicates a differentiation in olfactory perception of odors in the life history of two sexes (Grabe et al., 2016). For example, in leaf-cutting ants, the workers have one enlarged glomerulus, similar to a macroglomerulus, located at the entrance of the antennal nerve into the antennal lobe, which is involved in detecting the trail pheromones (Kleineidam et al., 2005; Kuebler et al., 2010). In honeybee males (drones), the enlarged glomerulus, MG2, respond specifically to the queen pheromone (9-ODA) while the plant odors are processed in other ordinary glomeruli (Sandoz, 2006).

In *B. dorsalis*, males can be strongly attracted to methyl eugenol (ME), but females do not show preference for this odor (Metcalf et al., 1975; Liu et al., 2017). This behavioral difference may be due to ME serving as a precursor for synthesis of the sex pheromone (Shelly et al., 2010; Tan and Nishida, 2012). Those glomeruli devoted to processing sex pheromone may be activated by its precursor, ME. The enlarged glomeruli in male AL are likely specialized for processing sex pheromones, which are glomeruli AM2, C1, L2, and L3, with the volume of AM2 being much larger than that of C1, L2, and L3 (Figure 6A). We therefore hypothesize that the glomerulus AM2 is involved in detection of ME. However, this needs to be physiologically verified. Male insects often have enlarged glomeruli to process information on female sex pheromones. Females, on the other hand, need to find suitable oviposition sites that are usually associated with plants. The identification of sexually dimorphic glomeruli in *B. dorsalis* from this study can certainly facilitate discovering important semiochemicals in this pest.

### Glomerular innervation pattern

The anterograde staining results show that the ascending axons from antenna or maxillary palps innervate not just the ipsilateral glomeruli but also contralateral glomeruli crossing the midline through the antennal commissure. The innervation pattern is symmetric, meaning that the OSNs from ipsilateral antennae or maxillary palp innervate the homologous glomeruli in both ALs. A similar projection pattern has also been found in the fruit fly (Stocker et al., 1990), medfly (Solari et al., 2016), mosquito (Anton et al., 2003; Ignell et al., 2005; Ghaninia et al., 2007), supporting a notion that contralateral projection of the sensory fibers is characteristic of dipterans. Morphologically, the bilateral innervation pattern derives from afferent branching, i.e., the same afferent fiber bifurcates with one branch innervating ipsilateral glomerulus and the other innervating the contralateral glomerulus (Stocker et al., 1990; Anton and Homberg, 1999). The functional advantages of such a branching pattern are still being actively investigated. One explanation is that the bilateral projection could enhance the signal-to-noise ratio during odor plume detection. For example, flies need bilateral sensory input to track odor gradients in flight (Duistermars et al., 2009). In *D. melanogaster* larvae, bilateral olfactory sensory input can enhance the ability to detect odors (Louis et al., 2008). However, as in *Drosophila*, not all glomeruli are bilaterally innervated (Figure 7). We found that five glomeruli only receive ipsilateral antennal input. These glomeruli are located at the lateral and posterior region of the AL (Figures 7E,F). It would be interesting to find out what qualities of odors are limited to ipsilateral processing and what requires bilateral processing. In *D. melanogaster*, five glomeruli located at lateral and posterior AL, VL1, V, VP1, VP2, and VP3 receive only ipsilateral projections (Stocker et al., 1983). Glomerulus V and VL1 receive the afferents from antennal Sensillum basiconic and coeloconic respectively, while VP2 and VP3 innervated by arista neurons (Stocker et al., 1983; Couto et al., 2005; Münch and Galizia, 2016).

Another interesting feature is that AL also receives afferents from the maxillary palps (Figures 3, 7). In total, we observed nine maxillary palp associated glomeruli (Figures 3H,I), and they are all bilaterally innervated. There is no overlap between antenna-linked and palp-linked glomeruli, suggesting that antenna and maxillary palp may supply parallel streams of olfactory information to AL. Similar organization was also found in fruit flies (Stocker, 1994), mosquitoes (Ignell et al., 2005; Ghaninia et al., 2007; Jung et al., 2015), and moths (Varela et al., 2009; Løfaldli et al., 2010; Zhao et al., 2016). In *D. melanogaster*, both antenna and maxillary palp contain Sensillum basiconica, which serves olfactory function (Stocker, 1994; de Bruyne et al., 1999, 2001). Particularly, in *Anopheles gambiae*, the ORNs from maxillary palps are highly sensitive to CO_2_ and 1-octen-3-ol (Lu et al., 2007). It is currently unknown what specific signal is detected by the maxillary palp of *B. dorsalis*.

### Antennal lobe tracts in *B. dorsalis*

In insects, the processed olfactory information is transmitted to the protocerebrum from antennal lobes in a parallel fashion via multiple ALTs. Three main tracts, i.e., medial, mediolateral, and lateral tracts, have been identified in honeybee (Kirschner et al., 2006), fruit fly (Stocker et al., 1990; Tanaka et al., 2012b; Ito et al., 2014), moth (Homberg et al., 1988), and ants (Zube and Rössler, 2008), in addition to some minor tracts. For the first time, we described the three typical ALTs in *B. dorsalis*, among which the mALT is the most prominent tract (Figure 8A). In addition, we also observed the transverse ALT (tALT) (Figures 8B,F), which was recently described in the noctuid moth, *Heliothis virescens* (Ian et al., 2016) and *D. melanogaster* (Tanaka et al., 2012a,b). The tALT deviates from the mALT, and then divides into two branches with one branch targeting the ipsilateral calyx and lateral horn and the other branch extending toward the posterior lateral protocerebrum (PLP). The staining in PLP in our preparation is too weak to be fully characterized. The accessory calyx (ACa) receives no innervations from ALTs (Figures 8F,G), supporting the idea that ACa is a center for processing visual information while Ca is a center for processing olfactory signals. In *Drosophila*, Kenyon cells responding to visual stimulation innervate ACa (Vogt et al., 2016).

The mALT (iACT in previous fruit fly studies) is the medial most pathway and contains the largest number of PNs (Wong et al., 2002; Tanaka et al., 2004). In our study, from the retrograde labeling from the calyces, the mALT can be visualized (Figure 9A).The dendritic ramifications of mALT projection neurons are separated into two roots, the dorsal and the ventral root. The cell bodies of the PNs in the ventral root are located in the posterior lateral to the AL while the dorsal root cell bodies are separated into two clusters, one in the anterodorsal side of the AL and other in the lateral AL. We infer that the dorsal and the ventral root is equivalent to the AL-mPN1 and -mPN4 tract, respectively (Tanaka et al., 2012a), due to the fact that first, the cell body positions are similar; second, they all have similar assembly patterns. In *Drosophila*, AL-mPN1 and -mPN4 tracts are all project via the mALT, and terminate in both the MB calyx and the LH. AL-mPN1s are classic unilateral uniglomerular neurons that arborize in a single glomerulus in the AL (Stocker et al., 1990; Wong et al., 2002; Tanaka et al., 2012a), while AL-PN4s are multiglomerular projections without forming any glomerular arborizationin the AL (Lai et al., 2008; Tanaka et al., 2012a). In this study, the location of the cell bodies of mALT neurons was labeled, which provides an accurate reference for the future use of intracellular recording or calcium imaging technology to study the function of PNs.

Our study points out similarities between *B. dorsalis* and *D. melanogaster* in their olfactory system. This is probably related to the phylogenetic relationship of these two species. *B. dorsalis* belongs to Tephritidae, while *D. melanogaster* branches from Drosophilidae, both being members of the Acalyptrata subsection of Brachyceran Diptera (Jacob et al., 2017). There is evidence that the antennal morphology and function of Tephritids and Drosophila are mostly conserved (Jacob et al., 2017). This is consistent with our observation that *B. dorsalis* and *D. melanogaster* share structural commonalities in their primary olfactory center. First, the number of glomeruli is similar - 64-65 in *B. dorsalis* and 56 in *D. melanogaster* (Tanaka et al., 2012a). Second, they all have non-overlapped glomeruli specialized for processing information from the antennae and the maxillary palps. Third, OSNs project bilaterally to both antennal lobes. Finally, they have the same number and type of antennal lobe tracts. All these suggest a close phylogenetic relationship between these two species.

## Author contributions

HL, XZ, BS, and TL for research direction and experimental design. TL and CL for acquisition of data. TL, HL, and JL for analysis and interpretation of data. TL and HL for writing the manuscript. TL, HL, BS, and XZ for revising for publication. XZ for research funding.

### Conflict of interest statement

The authors declare that the research was conducted in the absence of any commercial or financial relationships that could be construed as a potential conflict of interest.

## Supplementary material

Supplementary Video 1 Time lapse of consecutive frontal sections of a male oriental fruit fly AL with synapsin labeling. Obtained from the same confocal stack as Figure 2. Scale bar = 25 μm. z step = 0.8 μm.

Supplementary Presentation 1 Interactive 3D AL atlas of a male oriental fruit fly. This file can be opened by the Amira (version 5.4 or higher) software, and the name and corresponding location of each glomerulus can be viewed by using the “Surface Editor” module in Amira.
